# Relationship between oral hypofunction and salivary biomarkers in older adults: a cross-sectional study

**DOI:** 10.1186/s12903-024-04556-4

**Published:** 2024-07-06

**Authors:** Kenji Masutomi, Mika Bando, Yuji Inagaki, Rie Kido, Yuta Uemura, Yukari Hatada, Jun-ichi Kido, Makoto Fukui, Daisuke Hinode, Hiromichi Yumoto

**Affiliations:** 1 Department of Periodontology and Endodontology, Tokushima University Graduate School of Biomedical Sciences, 3-18-15 Kuramoto-Cho, Tokushima, 770-8504 Japan; 2 Department of Hygiene and Oral Health Science, Tokushima University Graduate School of Biomedical Sciences, 3-18-15 Kuramoto-Cho, Tokushima, 770-8504 Japan

**Keywords:** Periodontitis, Oral hypofunction, Saliva, Biomarkers, Older adults

## Abstract

**Background:**

Oral health problems have increased among older adults. Oral hypofunction is characterized by seven signs and symptoms: oral uncleanness, oral dryness, decline in occlusal force, decline in the movement function of the tongue and lips, decline in tongue pressure, decline in masticatory function, and decline in swallowing function, the latter being a significant risk factors for oral frailty. Recent research has suggested that salivary biomarkers can be used to assess not only oral diseases, including dental caries and periodontitis, but also systemic diseases, such as cancer and diabetes mellitus. This cross-sectional study investigated the relationship between oral hypofunction and the levels of salivary biomarkers.

**Methods:**

In total, 116 patients, aged 65 years or older, were included in this cross-sectional study. If three or more signs or symptoms in seven kinds of tests met the criteria of each test, oral hypofunction was diagnosed. The levels of biomarkers in the saliva collected from the patients were analyzed using an enzyme-linked immunosorbent assay.

**Results:**

In total, 63.8% of patients were diagnosed with oral hypofunction. Multivariable linear regression analysis showed that calprotectin levels in the saliva were significantly related to oral moisture and masticatory function. Furthermore, 8-OHdG levels in saliva were associated with the movement function of the tongue and lips and oral hygiene level, and salivary AGE correlated only with the movement function of the tongue and lips. Multiple logistic regression analysis revealed that calprotectin levels in the saliva were significantly correlated with the prevalence of oral hypofunction, even after adjusting for age, sex, and periodontal status. However, none of the biomarker levels in the saliva had a significant relationship with the number of examinations outside the reference range.

**Conclusions:**

Calprotectin, 8-OHdG, and AGE levels are associated with oral hypofunction in older adults.

**Supplementary Information:**

The online version contains supplementary material available at 10.1186/s12903-024-04556-4.

## Background

Frailty is a condition in which physical and mental vulnerability develops with age, significantly impacting the health and quality of life of older adults [1–3]. It represents a state of vulnerability to adverse outcomes, including functional decline, hospitalization, disability, long-term care, and death [4, 5]. Various risk factors for frailty, such as underlying diseases, nutritional status, and sarcopenia, interact with each other to increase vulnerability [6]. Various cross-sectional studies have reported an association between the number of teeth and frailty in older adults [7–9].

Oral frailty is a new concept employed as a preventive strategy to maintain or improve the eating/chewing ability of older adults in Japan, affecting food intake diversity, appetite, and nutritional intake associated with physical frailty [10]. Some papers have reported on the association between poor oral health conditions and frailty, using oral frailty index-8 or the Geriatric Oral Health Assessment Index as indicators of oral frailty [11, 12]. The relationship between oral frailty, including oral health status deterioration; deterioration of oral motor skills, saliva disorders; and oral pain, and frailty has been reported not only in Japan but also worldwide [13]. Accordingly, the recovery from oral frailty is important not only for maintaining oral hygiene to prevent periodontitis and dental caries but also for restoring function by providing appropriate prostheses for tooth loss.

In 2016, the Japanese Society of Gerodontology defined “oral hypofunction” as the state in which more than three of seven oral functional measures (poor oral hygiene, oral dryness, reduced occlusal force, decreased movement function of the tongue and lips, decreased tongue pressure, decreased masticatory function, and deterioration of swallowing function) met the diagnostic criteria [14]. By 2025, it is estimated that the population aged > 65-years old in Japan will reach 36,770,000 people. Even as the population aged 65 years and older declines after 2042, the aging rate will continue to rise, reaching 38.4% by 2065, when one out of every 2.6 persons in Japan will be aged 65 or older [15]. Medical, dental, and nursing care support are essential for the major phenomenon of a super-aged society. Since 2018, the Japanese government has adopted the concept of oral frailty as an important criterion for oral hypofunction in the glossary of disease diagnoses in the national dental insurance program. However, evaluation methods for oral hypofunction are of different types, require medical instruments and equipment, and are very complex.

Numerous systemic diseases are diagnosed using various biological molecules in body fluids. Saliva has multiple functions, including mouth cleaning, protection, antibacterial effects, and digestion. Saliva, as a non-invasive, safe, and simple source, could be useful for diagnosing oral diseases (i.e., dental caries, periodontitis, and oral cancer), as well as systemic conditions (i.e., diabetes mellitus, cardiovascular disease, and cancer, or Sjögren’s syndrome) [16–18].

Calprotectin is a calcium-binding protein typically produced by granulocytes, monocytes, macrophages, and epithelial cells [19]. Calprotectin levels in the plasma, synovial fluid, and feces are increased in some inflammatory diseases, such as cystic fibrosis [20], rheumatoid arthritis [21], ulcerative colitis [22], and Alzheimer’s dementia [23]. It has been reported that salivary calprotectin levels are increased in patients with periodontitis, oral candidiasis, or Sjögren’s syndrome but decreased in human immunodeficiency virus infection [24, 25].

8-Hydroxyguanosine (8-OHdG) is an oxidative stress biomarker and an oxidized nucleoside that is excreted in body fluids as a reparative consequence of DNA. A stable product is formed as a result of enzymatic dissolution after reactive oxygen species induce the 8-hydroxylation of guanine based on mitochondrial and nuclear DNA [26, 27]. Salivary 8-OHdG levels are recognized as oxidative biomarkers of diabetes mellitus [28] and periodontitis [29]. Furthermore, a systematic review reported that frailty and pre-frailty appear to be associated with higher oxidative stress and higher levels of 8-OHdG [30].

Elevated oxidative stress also promotes the formation of the advanced glycation end product (AGE) [31]. AGE levels are correlated with aging and the onset and exacerbation of a variety of diseases, including diabetes mellitus, atherosclerosis, and Alzheimer’s disease [32–35].

It has been reported that inflammatory markers, elevated interleukin-1β (IL-1β), or detectable C-reactive protein (CRP) in GCF are correlated with low levels of leisure-time physical activity [36]. However, there are no reports describing the association between salivary biomarkers, other than inflammatory markers, and oral hypofunction or oral frailty.

In this study, we investigated the relationship between oral hypofunction and salivary biomarkers such as calprotectin, 8-OHdG, and AGE in older adults, aiming to identify salivary biomarkers associated with oral hypofunction.

## Methods

### Clinical Study design and participants

This cross-sectional study was conducted in accordance with the Declaration of Helsinki and the STROBE Statement, was approved by the Ethics Committee of the Tokushima University Hospital (approval number: 3729). Informed consent was obtained from a total of 116 patients, either for first time or undergoing treatment, 18 of whom came to the Department of Periodontology at the Tokushima University Hospital and 98 of whom came to Masutomi Dental Clinic between April 2020 and December 2022. The inclusion criteria were patients aged ≥ 65 years with > 10 teeth, and the exclusion criteria were patients with severe systemic diseases (such as history of cerebrovascular accident with residual paralysis, rheumatoid arthritis, or patients undergoing cancer treatment). Patients were interviewed regarding other systemic medical histories (diabetes mellitus, hypertension, and heart disease). The % level of hemoglobin A1c (HbA1c) or years of history with diabetes mellitus were checked by the results of blood tests at medical checkups or diabetic data book for self-management. 38 patients had no test results, and test values could not be verified.

### Evaluation of periodontal status

All periodontal variables were assessed at six sites (mesiobuccal, midbuccal, distobuccal, mesiolingual, midlingual, and distolingual) per tooth for all residual teeth. The clinical parameters recorded were plaque control record, probing pocket depth (PD), and percentage of bleeding at the probing sites (BOP%). The percentage of site with PD 4 mm or more in all measured sites was denoted as PD ≥ 4 mm (%), and PD > 6 mm (%) is also denoted in same way. Periodontitis status was classified according to the guidelines of the world workshop conducted jointly by the American Academy of Periodontology and the European Federation of Periodontology in 2017 [37].

### Examinations of oral hypofunction and systemic frailty

Examination of oral hypofunction with respect to oral hygiene, oral moisture, occlusal force, movement function of the tongue and lips, tongue pressure, masticatory function, and swallowing function was performed according to previously described methods [14]. Oral hypofunction was diagnosed when the scores in more than three of these seven tests exceeded the reference value.

Oral hygiene was examined by measuring the number of bacteria collected from the tongue dorsum with a cotton swab using a bacterial quantitative analysis device (bacterial counter, Panasonic, Osaka, Japan) and assessed from levels 1 to 7. Oral hygiene was considered poor when the level was > 4. The total number of microorganisms 3.162 × 10^6^ CFU/ mL or higher is considered Level 4 or higher.

The oral moisture test was performed by measuring oral mucosal wetness at the center of the tongue dorsum using an oral moisture meter (Mucus, Life Co. Ltd., Saitama, Japan). Oral dryness was diagnosed when the average score from the three tests was < 27.

Occlusal force was measured using a pressure-sensitive sheet (Dental Prescale II, GC, Tokyo, Japan) during clenching for 3 s in the occlusal position, and a decline in occlusal force was diagnosed when the score was < 500 N. If participants wore dentures, the test was performed with the dentures in place.

The movement function of the tongue and lips was assessed by counting the instances participants pronounced three monosyllables (“Pa,” “Ta,” and “Ka,”) for 5 s using an automatic measuring device (Kenkou-kun Handy, Takei Kikai Kogyo, Niigata, Japan) and the decline of movement function of the tongue and lips was diagnosed when this score was < 6 times per second.

Tongue pressure was measured using a tongue pressure-measuring device (JMS Tongue Depressor; GC, Tokyo, Japan). A probe with a balloon-shaped tip was placed between the tongue and palate, and the pressure of crushing with the tongue was measured. A decline in tongue pressure was diagnosed when the value was < 30 kPa.

A glucose analyzer (Glucosensor GS-II, GC, Tokyo, Japan) was used for the masticatory function test. The glucose concentration in the saliva was determined by chewing gum containing 2 g of glucose for 20 s. A decline in masticatory function was diagnosed when the glucose concentration in the saliva was < 100 mg/dL.

The swallowing function test was performed using a subjective swallowing screening questionnaire (10-item Eating Assessment Tool [EAT-10]) [38]. A decline in swallowing function was diagnosed when more than three test items were checked.

The systemic frailty test was performed using a checklist developed by the Ministry of Health, Labor, and Welfare. Systemic frailty was diagnosed when > 10 items were assessed [39].

### Saliva sampling

Participants were instructed to refrain from brushing their teeth, drinking, and eating within 1 h before saliva sampling according to the conditions described in the saliva test kit instructions (Saliva Check Lab, GC, Tokyo, Japan). Stimulated whole saliva was collected. Participants chewed tasteless and odorless gum in the kit for 5 min, and whole saliva was deposited into the collection cup. Part of the collected saliva was transferred into a container at the Saliva Check Lab and sent to the GC Oral Check Center, where the proportion of *Porphyromonas gingivalis* to total number of bacteria in the saliva was determined by real-time polymerase chain reaction. Remained saliva was centrifuged at 10,000 × g for 10 min at 4 °C to remove debris and stored at ₋80 °C for enzyme-linked immunosorbent assay (ELISA).

### Measurement of biomarkers

The concentrations of calprotectin, 8-OHdG, and AGE in the saliva were determined using ELISA kits (Human Calprotectin ELISA Kit, Hycult Biotechnology, Uden, Netherlands; Human 8-OHdG ELISA Kit, Japan Institute for the Control of Aging, NIKKEN SEIL Co, Ltd. Shizuoka, Japan; and Oxiselect™ Advanced Glycation End Product Competitive ELISA Kit, Cell Biolabs, Inc., San Diego, CA) according to the manufacturer’s instructions, and expressed as nanograms per milliliter of saliva.

### Statistical analysis

Sex, oral hypofunction, periodontal status, hypertension, heart disease, and diabetes mellitus, treated as categorical variables, were presented as number of patients or percentage, and all other variables treated as continuous variables were indicated as median (Interquartile range:25th-75th). The Mann–Whitney U test was used to compare continuous variables, and the Chi-square test was used to compare categorical variables. A simple correlation analysis was performed using Spearman’s rank correlation coefficient. Multiple logistic regression analysis was conducted to explore the relationship between oral hypofunction and each oral biomarker (calprotectin, 8-OHdG, and AGE). The diagnosis of oral hypofunction (0, normal oral function; 1, oral function) was used as objective variable, and each oral biomarker concentration was used as explanatory variables. Additionally, multivariable linear regression analysis was performed to examine the relationship between the number of examinations outside the reference range and each salivary biomarker (calprotectin, 8-OHdG, and AGE). In this analysis, the number of examinations outside the reference range was used as objective variables, and a natural logarithmic transformation of each salivary biomarker concentration to approximate a normal distribution was used as explanatory variables. For the evaluation of the correlation between the biomarkers in saliva and oral hypofunction test values, the multivariable linear regression analysis was performed with a natural logarithmic transformation of each saliva biomarker as objective variables and each oral hypofunction test value as explanatory variables. Multiple logistic regression analysis and multivariable linear regression analysis were performed with covariate-adjusted for age and sex (Model 1) or age, sex and periodontal stage (Model 2) as predictors and confounders. All analyses were performed with the maximum number of missing values excluded. There analyses were conducted using EZR (Saitama Medical Center, Jichi Medical University, Saitama, Japan), a graphical user interface for a modified version of R commander (The R Foundation for Statistical Computing, Vienna, Austria), which includes statistical functions frequently used in biostatistics. Statistical significance was set at a *P* value < 0.05.

## Results

### Characteristics of participants

In total, 116 participants (median age 73.0) were enrolled in this study. Table 1 lists the basic information of the participants, prevalence of oral hypofunction, number of participants with periodontitis status III + IV, and values for all oral examinations. There were fewer male 40 (34.5%) than females 76 (65.5%). The number and prevalence of patients with oral hypofunction and periodontitis status III + IV were 74 (63.8%) and 70 (60.3%), respectively, accounting for approximately half of participants. Table 2 contains a comparison of the characteristics of patients with normal oral function and those with oral hypofunction. Participants diagnosed with oral hypofunction had significantly poor oral hygiene, oral dryness, reduced occlusal force, reduced movement function of the tongue and lips, and decreased tongue pressure than those in the normal oral function group (*P* < 0.01). However, there was no significant difference between the normal oral function group and oral hypofunction group in terms of decreased masticatory function (*P* = 0.55) or swallowing function (*P* = 0.081). The morbidity of any systemic disease and rate of periodontitis stage classified as III + IV did not differ between the two groups.

**Table 1:** Clinical characteristics of participants enrolled in this study

	Median (25^th^—75^th^) **Number (%)**
Participants	116
Age (years)^a^	73.0 (70.0—77.0)
Sex (male)^b^	40 (34.5)
Remaining teeth^a^	23.5 (19.3—26.0)
Diabetes mellitus^b^	41 (35.3)
Hypertension^b^	60 (51.7)
Heart disease^b^	15 (12.9)
Oral hypofunction^b^	74 (63.8)
The number of examinations outside the reference range^a^	3.0 (2.0—4.0)
Oral hygiene level^a^	5.0 (4.0—5.0)
Oral moisture^a^	27.8 (26.0—29.3)
Occlusal force (N)^a,c^	734.7 (506.7—947.3)
Movement function of the tongue and lip (times/second)	
/Pa/^a^	6.0 (5.6—6.4)
/Ta/^a^	6.0 (5.3—6.4)
/Ka/^a^	5.6 (5.1—6.0)
Tongue pressure (kPa)^a^	30.1 (25.5—34.6)
Masticatory function (mg/dL)^a^	233.5 (196.3—276.0)
Swallowing function (score)^a^	0 (0.0—1.0)
Systemic frailty (score)^a^	3.0 (2.0—5.0)
Periodontitis stage III + IV^b^	70 (60.3)
PD ≥ 4 mm (%)^a,d^	18.1 (9.3—29.4)
PD ≥ 6 mm (%)^a,d^	1.5 (0—5.6)
PD ≥ 6 mm (site)^a,d^	2.0 (0 -7.0)
BOP (%)^a,d^	18.5 (10.1 -31.5)
Proportion of *P. gingivalis* (%)^a^	0.007 (0—0.034)

^a^Data are expressed as median (25th—75th)

^b^Data are expressed as number (percentage)

^c^7 missing values exist in data

^d^1 missing value exists in data

**Table 2:** Comparison of characteristics between paticipants with normal oral function and those of oral hypofunction

	Median (25^th^—75^th^) **Number (%)**		
	Normal oral function (*n* = 42)	Oral hypofunction (*n* = 74)	*P*-value
Age (years)^a^	72.0 (70.0—75.5)	74.0 (70.0—78.0)	0.43
Sex (male)^b^	19 (45.2)	21 (28.4)	0.072
Remaining teeth^a^	24.0 (21.0—27.0)	23.0 (18.0—26.0)	0.069
Periodontitis stage III + IV^b^	23 (54.8)	47 (63.5)	0.43
Diabetes mellitus^b^	41 (35.3)	28 (37.8)	0.55
Hypertension^b^	60 (51.7)	41 (55.4)	0.34
Heart disease^b^	15 (12.9)	11 (14.9)	0.57
The number of examinations outside the reference range^a^	2.0 (1.0—2.0)	3.0 (3.0—4.0)	< 0.001*
Oral hygiene level (≥ 4)^b^	28 (66.7)	68 (91.9)	0.002*
Oral moisture (≤ 27)^b^	9 (21.4)	39 (52.7)	0.002*
Occlusal force (≤ 500 N)^b,c^	3 (7.5)	23 (33.3)	0.002*
Movement function of the tongue and lip (any counts of Pa, Ta, Ka ≤ 6 times/second)^b^	22 (52.4)	64 (86.5)	< 0.001*
Tongue pressure (≤ 30 kPa)^b^	3 (7.1)	54 (73.2)	< 0.001*
Masticatory function (≤ 100 mg/dL)^b^	0 (0)	3 (4.2)	0.55
Swallowing function (≥ 3 score)^b^	2 (4.8)	12 (16.2)	0.081
Systemic frailty (≥ 10 score)^a^	3.0 (1.0—4.3)	4.0 (2.0—5.0)	0.14

Statistical analysis was performed using Mann–Whitney U test or Chi-square test

^a^Data are expressed as median (25th—75th)

^b^Data are expressed as number (percentage)

^c^7 missing values exist in data; 2 in Normal oral function group and 5 in Oral hypofunction group

^*^P < 0.05

### Correlation between biomarkers in saliva and clinical variables

Table 3 presents the results of the simple correlation analysis between each biomarker in saliva and clinical variables derived from diabetes mellitus parameters, oral hypofunction tests, and periodontal index. The concentration of calprotectin in saliva exhibited weak correlations with movement function of the tongue and lips (/Ta/*r* = 0.19, *P* = 0.047), oral moisture (*r* = -0.23, *P* = 0.014), and masticatory function (*r* = 0.27, *P* = 0.036). Weak negative correlations were identified between the concentration of 8-OHdG in saliva and the movement of the tongue and lips (/Pa/ *r* = -0.20, *P* = 0.034, /Ta/ *r* = -0.27, *P* = 0.004, /Ka/ *r* = -0.22, *P* = 0.018), as well as masticatory function (*r* = -0.19, *P* = 0.041). Additionally, weak positive correlations were observed with HbA1c (most recent% *r* = 0.39, *P* < 0.001, max% *r* = 0.43, *P* < 0.001), and the duration of diabetes mellitus (*r* = 0.40, *P* < 0.001). However, for HbA1c, the simple correlation analysis was performed on the data of 78 participants whose test values were available. Regarding the concentration of AGE, a negative correlation was observed between the movement function of the tongue and lips (/Pa/ *r* = -0.22, *P* = 0.017; /Ta/ *r* = -0.23, *P* = 0.013; /Ka/ *r* = -0.20, *P* = 0.03), whereas positive correlations were observed with the number of examinations outside the reference range (*r* = 0.19, *P* = 0.043) and BOP% (*r* = 0.26, *P* = 0.005).

**Table 3:** Simple correlation analysis between biomarkers in saliva and clinical variables

	**calprotectin** **(ng/ml)**		**8-OHdG** **(ng/ml)**		**AGE** **(ng/ml)**	
	*r*	*P*-value	*r*	*P-*value	*r*	*P-*value
Age (years)	0.18	0.063	0.0725	0.44	0.122	0.20
Remaining teeth	-0.018	0.85	-0.0536	0.57	0.00787	0.93
Diabetes mellitus parameters						
・HbA1c (most recent %)^c^	0.063	0.59	0.39	< 0.001*	0.21	0.073
・HbA1c (max %)^c^	-0.085	0.47	0.43	< 0.001*	0.22	0.055
・Duration (years)^d^	-0.048	0.62	0.40	< 0.001*	0.084	0.38
The number of examinations outside the reference range	0.056	0.56	0.14	0.13	0.19	0.043*
Oral hygiene level	-0.025	0.79	-0.13	0.17	-0.13	0.17
Oral moisture	-0.23	0.014*	0.052	0.58	-0.070	0.46
Occlusal force (N)^a^	0.044	0.66	-0.044	0.65	-0.051	0.60
Movement function of the tongue and lip (times/second)						
/Pa/	-0.023	0.81	-0.20	0.034*	-0.22	0.017*
/Ta/	0.19	0.047*	-0.27	0.004*	-0.23	0.013*
/Ka/	0.18	0.059	-0.22	0.018*	-0.20	0.03*
Tongue pressure (kPa)	-0.002	0.99	-0.025	0.80	0.006	0.95
Masticatory function (mg/dL)	0.27	0.036*	-0.19	0.041*	-0.088	0.35
Swallowing function (score)	0.010	0.30	-0.007	0.94	-0.082	0.38
Periodontal index						
・PD ≧4 mm (%)^b^	0.0029	0.98	0.055	0.57	0.18	0.053
・PD ≧6 mm (%)^b^	-0.019	0.84	0.072	0.45	0.069	0.46
・PD ≧6 mm (sites)^b^	-0.017	0.86	0.065	0.49	0.085	0.37
・BOP (%)^b^	-0.0075	0.94	0.132	0.16	0.26	0.005*
* ・*Proportion of *P. gingivalis (%)*	0.044	0.65	-0.029	0.76	0.071	0.45
Systemic frailty (score)	0.010	0.30	0.14	0.14	0.10	0.28

Statistical analysis was performed using Spearman’s rank correlation coefficient

^a^7 missing values exist in data

^b^1 missing value exists in data

^c^38 missing values exists in data

^d^2 missing values exists in data

^*^P < 0.05, *r*: correlation coefficient

Table 4 lists the results of the multivariable linear regression analysis for the association of each biomarker in saliva and each oral hypofunction test value with covariate-adjusted for age and sex (Model 1) or age, sex and periodontal stage (Model 2) as confounders. Calprotectin level in saliva was significantly related to oral moisture (Model 1; *P* = 0.01, Model 2; *P* = 0.013) and masticatory function (Model 1; *P* = 0.006, Model 2; *P* = 0.007). Furthermore, 8-OHdG level in saliva was associated with oral hygiene (Model 1; *P* = 0.05, Model 2; *P* = 0.05) and the movement function of the tongue and lip (Model 1; /Ta/ *P* = 0.031, Model 2; /Ta/ *P* = 0.03, /Ka/ *P* = 0.048). The association with masticatory function, which was correlated with 8-OHdG in Table 3, also showed a trend, although not significantly (Model 1; *P* = 0.077, Model 2; *P* = 0.085). AGE level in saliva was significantly related to only the movement function of the tongue and lip (Model 1; /Ta/ *P* = 0.04, Model 2; /Ta/ *P* = 0.037).

**Table 4:** Multivariable linear regression analysis with biomarkers in saliva

**a** calprotectin (*n* = 107)								
		Model 1				Model 2		
Expalanatory Variables	Estimate	95% CI		*P*-value	Estimate	95% CI		*P*-value
Oral hygiene level	0.006	-0.169	0.181	0.946	0.007	-0.169	0.183	0.933
Oral moisture	-0.096	-0.168	-0.023	0.01*	-0.094	-0.167	-0.02	0.013*
Occlusal force (*N*)	-8.7E-05	0	0	0.664	-9.6E-05	0	0	0.633
Movement function of the tongue and lip (times/second)								
/Pa/	0.032	-0.179	0.243	0.764	0.038	-0.174	0.249	0.726
/Ta/	0.173	-0.041	0.387	0.112	0.173	-0.041	0.388	0.112
/Ka/	0.087	-0.111	0.284	0.386	0.093	-0.105	0.292	0.352
Tongue pressure (kPa)	-0.005	-0.033	0.023	0.715	-0.006	-0.034	0.022	0.678
Masticatory function (mg/dL)	0.003	0.001	0.006	0.006*	0.003	0.001	0.006	0.007*
Swallowing function (score)	-0.001	-0.069	0.068	0.984	0.003	-0.066	0.073	0.928
**b** 8-OHdG (*n* = 108)								
		Model 1				Model 2		
Expalanatory Variables	Estimate	95% CI		*P*-value	Estimate	95% CI		*P*-value
Oral hygiene level	-0.125	-0.249	0	0.05*	-0.125	-0.25	0	0.05*
Oral moisture	0.017	-0.037	0.071	0.528	0.015	-0.04	0.069	0.596
Occlusal force (*N*)	-5.3E-05	0	0	0.724	-4E-05	0	0	0.79
Movement function of the tongue and lip (times/second)								
/Pa/	-0.131	-0.282	0.02	0.089	-0.136	-0.288	0.016	0.079
/Ta/	-0.171	-0.326	-0.016	0.031	-0.172	-0.327	-0.017	0.03*
/Ka/	-0.14	-0.283	0.003	0.055	-0.145	-0.288	-0.002	0.048*
Tongue pressure (kPa)	0.005	-0.015	0.024	0.64	0.005	-0.014	0.025	0.597
Masticatory function (mg/dL)	-0.002	-0.003	0	0.077	-0.002	-0.003	0	0.085
Swallowing function (score)	-0.008	-0.06	0.044	0.765	-0.011	-0.063	0.042	0.688
**c** AGE (*n* = 109)								
		Model 1				Model 2		
Expalanatory Variables	Estimate	95% CI		*P*-value	Estimate	95% CI		*P*-value
Oral hygiene level	-0.217	-0.612	0.178	0.279	-0.222	-0.61	0.166	0.259
Oral moisture	-0.027	-0.197	0.142	0.752	-0.053	-0.221	0.115	0.529
Occlusal force (*N*)	0	-0.001	0.001	0.741	-3.6E-05	-0.001	0.001	0.938
Movement function of the tongue and lip (times/second)								
/Pa/	-0.342	-0.818	0.134	0.158	-0.384	-0.852	0.084	0.107
/Ta/	-0.507	-0.99	-0.024	0.04*	-0.505	-0.979	-0.031	0.037*
/Ka/	-0.244	-0.694	0.205	0.283	-0.288	-0.73	0.154	0.2
Tongue pressure (kPa)	0.003	-0.059	0.064	0.932	0.008	-0.053	0.069	0.79
Masticatory function (mg/dL)	-0.003	-0.008	0.003	0.342	-0.002	-0.008	0.003	0.418

Model 1 adjusted for age and sex (0 = female, 1 = male)

Model 2 adjusted for the variables in model 1 and periodontal stage (0 = stage I + II, 1 = stage III + IV)

*P*-value were derived using Multiple liner regression analysis

*P < 0.05, *CI* confidence interval

Objective variable was a natural logarithmic transfomation of each salivary biomarker concentration, calprotectin (a), 8-OHdG (b) and AGE (c)

All explanatory variables other than sex and periodontal stage were continuous variables

### Association between susceptibility to oral hypofunction and biomarkers in saliva

**Fig. 1:**
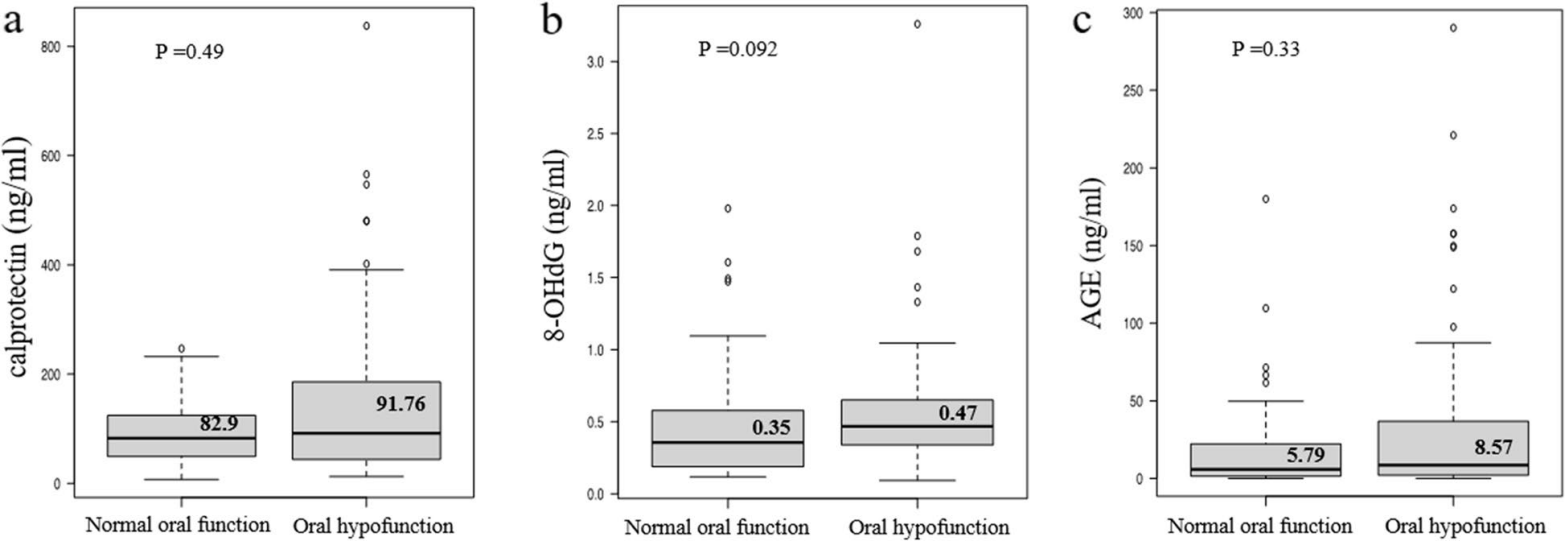
Biomarker levels in saliva of participants with normal oral function and oral hypofunction. Concentrations of (**a**) calprotectin, **b** 8-OHdG, and **c** AGE in the saliva of participants with normal and oral hypofunction. The median is presented in each box beard. Statistical analysis was performed using Mann–Whitney U test

Figure 1 illustrates a comparison of the levels of each biomarker in the saliva of participants with normal oral function and in those with oral hypofunction. No significant differences were found in any of the oral biomarker levels between the normal oral function and oral hypofunction groups. However, the median concentration in the oral hypofunction group was slightly higher than that in the normal oral function group.

To determine the relationship between the diagnosis of oral hypofunction and each biomarker in the saliva, multiple logistic regression analysis was performed after adjusting for covariates (Model 1: age and sex; and Model 2: age, sex, and periodontal stage). As shown in Table 5, the concentration of calprotectin in saliva was significantly associated with the diagnosis of oral hypofunction in Models 1 (*P* = 0.048) and 2 (*P* = 0.047). These significant associations between the concentration of calprotectin and the diagnosis of oral hypofunction were similar, even after adjusting for the prevalence of diabetes mellitus or hypertension (Additional file 1, Models 3 and 4). However, 8-OHdG, and AGE levels were not associated with oral hypofunction.

**Table 5:** Multiple logistic regression analysis with the diagnosis of oral hypofunction as the objective variable

	**Model 1**			**Model 2**		
Explanatory Variables	OR	95%Cl	*P*-value	OR	95%Cl	*P*-value
calprotectin	1.004	1.000–1.009	0.048*	1.004	1.000–1.009	0.047*
8-OHdG	1.294	0.490–3.414	0.603	1.300	0.500–3.382	0.590
AGE	1.005	0.994–1.016	0.340	1.005	0.995–1.016	0.331

Model 1 adjusted for age and sex (0 = female, 1 = male)

Model 2 adjusted for the variables in model 1 and periodontal stage (0 = stage I + II, 1 = stage III + IV)

*P*-value was derived using Multiple Logistic regression analysis. *P < 0.05, *OR* odds ratio, *CI* confidence interval

Objective variable was the diagnosis of oral hypofunction (Normal oral function = 0, Oral hypofunction = 1)

All explanatory other than sex and periodontal stage were continuous variables

### Correlation between the number of examinations outside the reference range and biomarkers in saliva

Table 6 shows the results of the simple correlation analysis between the number of examinations outside the reference range and clinical variables (diabetes mellitus parameters, oral hypofunction test values, periodontal disease status, and salivary biomarkers). All test values of oral hypofunction were significantly correlated with the number of examinations outside the reference range; occlusal force (*r* = -0.41, *P* < 0.001), movement function of the tongue and lips (/Pa/ *r* = -0.27, *P* = 0.003; /Ta/ *r* = -0.34, *P* < 0.001; /Ka/ *r* = -0.27, *P* = 0.003), and tongue pressure (*r* = -0.53, *P* < 0.001) were moderately correlated. The number of remaining teeth (*r* = -0.24, *P* = 0.009) and the concentration of AGE in the saliva (*r* = 0.19, *P* = 0.043) were weakly correlated with the number of examinations outside the reference range. There was no correlation between the diabetes mellitus parameters or periodontal disease status and the number of examinations outside the reference range.

**Table 6:** Simple correlation analysis between the number of examinations outside the reference range and clinical variables

	**The number of examinations outside the reference range**	
	*r*	*P-*value
Age (years)	0.046	0.63
Remaining teeth	-0.24	0.009*
Diabetes mellitus parameter		
HbA1c (most recent %)^c^	0.13	0.28
HbA1c (max %)^c^	0.10	0.36
Duration (years)^d^	-0.031	0.75
Oral hygiene (level)	0.19	0.042*
Oral moisture	-0.34	< 0.001*
Occlusal force (N)^a^	-0.41	< 0.001*
Movement function of the tongue and lip (times/second)		
/Pa/	-0.27	0.003*
/Ta/	-0.34	< 0.001*
/Ka/	-0.27	0.003*
Tongue pressure (kPa)	-0.53	< 0.001*
Masticatory function (mg/dL)	-0.24	0.011*
Swallowing function (score)	0.19	0.039*
Periodontal index		
PD ≧4 mm (%)^b^	0.067	0.48
PD ≧6 mm (%)^b^	0.063	0.51
PD ≧6 mm (sites)^b^	0.049	0.60
BOP (%)^b^	0.12	0.22
*P. gingivalis* (%)	0.15	0.10
Systemic frailty (score)	0.18	0.059
Biomarkers in saliva		
calprotectin (ng/ml)	0.056	0.56
8-OHdG (ng/ml)	0.14	0.13
AGE (ng/ml)	0.19	0.043*

^a^7 missing values exist in data

^b^1 missing value exists in data

^c^38 missing values exists in data

^d^2 missing values exists in data

*P*-value was derived using Spearman’s rank correlation coefficient

^*^P < 0.05, r: correlation coefficient

Table 7 lists the results of the multivariable linear regression analysis for the association of the number of examinations outside the reference range with each biomarker in the saliva. We found no significant associations between the number of examinations outside the reference range and the concentrations of biomarkers (calprotectin, 8-OHdG, and AGE) in Model 1 (adjusted for age and sex) and Model 2 (adjusted for age, sex, and periodontal stage), even after adjusting for the prevalence of systemic diseases (Additional file 2, Models 3 and 4).

**Table 7:** Multivariable linear regression analysis with the number of examinations outside the reference range

	**Model 1**			**Model 2**		
Explanatory Variables	Estimate	95%Cl	*P*-value	Estimate	95%Cl	*P*-value
calprotectin (*n* = 111)	0.13005	-0.11150, 0.3716	0.29	0.13945	-0.10230, 0.38119	0.26
8-OHdG (*n* = 114)	0.08556	-0.23661, 0.40773	0.60	0.07259	-0.25024, 0.39543	0.66
AGE (*n* = 115)	0.07499	-0.02675, 0.17673	0.15	0.06475	-0.03930, 0.16881	0.22

Model 1 adjusted for age and sex (0 = female, 1 = male)

Model 2 adjusted for the variables in model 1 and periodontal stage (0 = stage I + II, 1 = stage III + IV)

*P*-value were derived using Multiple liner regression analysis. CI: confidence interval

Objective variable was the number of examinations outside the reference range

All explanatory variables other than sex and periodontal stage were continuous variables

## Discussion

In this study, most participants had systemic disease, with hypertension being prevalent in 51.7%, diabetes mellitus in 35.3%, and heart disease in 12.9%. This study found that 63.8% of the participants experienced oral hypofunction. This prevalence is similar to the 63% reported in a previous study [40], which included 134 participants (81 females and 53 males) aged 32–93 years (mean age 75.2) with similar characteristics to our study. Examining the median values of oral function tests in our study, occlusal force and glucose concentration as masticatory function indicators were 734.7 N and 233.5 mg/dL, respectively. In contrast, another study reported values of 563.4 N and 132.6 mg/dL for these parameters. The high masticatory function observed in our study may be explained by the larger number of remaining teeth (23.5) compared to the 15.3 teeth reported in the previous study [40].

We found no significant difference in age between the normal oral function group and the oral hypofunction group (Table 2). This result differs from those of previous studies in which there was a significant difference in age between the two groups [40, 41]. The reason for this difference may be that the participants in these previous studies were between 32 and 92 years of age [40] or 20 years of age or older [41], whereas the participants in our study were 65 years of age or older. We did not find any difference in the rate of patients diagnosed with periodontitis III + IV between the two groups. Ramsay et al. [9] reported that the percentage of sites with PD > 3.5 mm (more than 20%) was not significantly correlated with the diagnosis of physical frailty. However, there is limited research on the association between periodontal disease status and oral hypofunction. Depending on the diagnosis of periodontal disease (extensive or localized) or the stage of the treatment for periodontal disease, the results may vary.

In this study, there was no significant difference in the number of remaining teeth between the normal oral function group and the oral hypofunction group (Table 2); however, the number of remaining teeth was weakly correlated with the number of examinations outside the reference range (Table 6). A previous study reported that people with 20 or more natural teeth were less susceptible to frailty than those with edentulism [42]. Furthermore, the number of remaining teeth, regardless of denture use, is significantly associated with the number of physical frailty phenotypes [12]. Our study included participants with 10 or more remaining teeth. This is because it has been reported that the risk of frailty is 1.7 times higher for participants with 1–10 teeth compared to those with 20 or more teeth [8], and also because of the impact of 20 or more teeth on the evaluation of occlusal force. Removing this limitation of remaining teeth might have revealed a more evident association between the number of remaining teeth and the diagnosis of oral hypofunction.

As observed in Table 2, masticatory and swallowing function were not associated with the diagnosis of oral hypofunction in our study. Hatanaka’s study [40] also showed oral hygiene and swallowing function were not associated with the diagnosis of oral hypofunction. These results suggest that criteria for the diagnosis of oral hypofunction and their reference value should be reconsidered by further studies on oral hypofunction.

For the first time, we investigated the utility of salivary biomarkers for the diagnosis of oral hypofunction. Figure 1 shows a comparison of salivary biomarker concentrations between participants with normal oral function and those with oral hypofunction. The median concentration of calprotectin in the normal oral function group was 82.9 ng/ml and 91.8 ng/ml in the oral hypofunction group. Mean calprotectin levels were approximately tenfold lower than those reported by Sweet et al. [25]. The median concentrations of 8-OHdG were 0.35 ng/ml and 0.47 ng/ml in the normal oral function group and the oral hypofunction group, respectively. These mean 8-OHdG levels are similar to those reported in a previous study [29] but were approximately tenfold lower than the results from another study [43]. The median concentrations of AGE were 5.79 ng/ml and 8.57 ng/ml in the normal oral function and oral hypofunction groups, respectively; however, few studies have examined AGE concentrations in saliva. The variations in the concentrations of these biomarkers across studies may be due to differences in saliva collection, processing methods, race, or age of the participants. Regarding all events in this study, the median concentrations in the oral hypofunction group were slightly higher than those in the normal oral function group.

There are no reports showing the association between those oral biomarkers and oral hypofunction or oral frailty. Simple correlation analysis found that calprotectin levels in saliva were weakly correlated with movement function of the tongue and lip, oral moisture, and masticatory function (Table 3). However, calprotectin level in saliva was significantly related to two oral hypofunctional tests of oral moisture and masticatory function in adjusting for covariate (age, sex and periodontal stage). Previous studies on the association between these biomarkers and oral or systemic diseases have reported that salivary calprotectin levels are elevated in patients with geographical tongue [44] or Shögren’s syndrome [25], suggesting a weakly correlation between salivary calprotectin and oral moisture via decreased salivation. Furthermore, the association between fecal calprotectin levels and Alzheimer's disease [23] and reports of calprotectin secretion from skeletal muscle tissue during exercise [45] suggest that calprotectin may be involved in movement function of the tongue and lip through its involvement in these muscles and neurotransmission. It is possible that decline of oral moisture and tongue and lip movement may also contribute to decreased masticatory function.

Salivary 8-OHdG levels were correlated with the movement function of the tongue and lip and masticatory function (Table 3). In multivariable linear regression analysis, 8-OHdG level in saliva was significantly related to oral hygiene and the movement function of the tongue and lip, and also showed a trend of association with masticatory function. The reported association between serum 8-OHdG concentration and sarcopenia [46], suggests that 8-OHdG may be related to movement function of the tongue and lip and masticatory function.

Salivary AGE was only correlated with the movement function of the tongue and lip. The accumulation of AGEs in skeletal muscle tissue with aging [47] and numerous reports on sarcopenia [48] suggest that AGEs may be related to movement function of the tongue and lip.

Multiple logistic regression analysis also showed that calprotectin levels in saliva were significantly associated with the prevalence of oral hypofunction, even after adjusting for age, sex, periodontal status, and systemic diseases (Table 5 and Additional file 1). Therefore, in this study, we demonstrated, for the first time, an association between salivary biomarkers and oral hypofunction. It may be difficult that the findings from this study lead to directly demonstrate the mechanism of association between biomarkers and oral hypofunction. However, accumulative results from future research to examine the relationship between each item of oral hypofunction and other biomarkers as well as further in vitro and in vivo studies may lead to elucidate the scientific mechanism of oral hypofunction. Moreover, these may be able to reduce the number of diagnostic items for oral hypofunction or to develop the simple diagnosis methods using immunochromatographic measurement devices etc. [49]. This suggests the possibility of non-invasive or simple examination for oral frailty or the development of frail prediction and prevention using saliva.

The present study has some limitations. First, causal relationships could not be clarified because this was a cross-sectional study. Therefore, a longitudinal study on patients diagnosed with oral hypofunction is required. Second, the sample size was small, limiting the extent and quality of the analysis. Third, the participants of this study were adults aged 65 years or older with at least 10 teeth; thus, the impact of age and remaining teeth on the diagnosis of oral hypofunction could not be examined. Finally, the participants were at different stages of periodontal therapy; therefore, it was not possible to critically determine the association between the periodontal index and the diagnosis of oral hypofunction or salivary biomarker levels.

## Conclusions

This is the first study to demonstrate that salivary biomarkers, especially calprotectin, are associated with the diagnosis of oral hypofunction in older adults.

### Supplementary Information

Additional file 1. Multiple logistic regression analysis with the diagnosis of oral hypofunction as the objective variable. *P*-value was derived using Multiple Logistic regression analysis. *P < 0.05, OR: odds ratio, CI: confidence interval. Objective variable was the diagnosis of oral hypofunction (Normal oral function = 0, Oral hypofunction = 1). All explanatory other than sex, periodontal stage, diabetes mellitus and hypertension were continuous variables. Model 1 adjusted for age and sex (0 = female, 1 = male). Model 2 adjusted for the variables in model 1 and periodontal stage (0 = stage I+II, 1 = stage III+IV). Model 3 adjusted for the variables in model 1 and diabetes mellitus (1 = positive). Model 4 adjusted for the variables in model 1 and hypertension (1 = positive).

Additional file 2. Multivariable linear regression analysis with the number of examinations outside the refence range. *P*-value was derived using Multiple liner regression analysis. CI: confidence interval. Objective variable was the number of examinations outside the reference range. All explanatory variables other than sex, periodontal stage and hypertension were continuous variables. Model 1 adjusted for age and sex (0 = female, 1 = male). Model 2 adjusted for the variables in model 1 and periodontal stage (0 = stage I+II, 1 = stage III+IV). Model 3 adjusted for the variables in model 1 and diabetes mellitus (1 = positive). Model 4 adjusted for the variables in model 1 and hypertension (1 = positive).

## Data Availability

The datasets used and/or analyzed during the current study are available from the corresponding author on reasonable request.

## Abbreviations

GCF: Gingival crevicular fluid
8-OHdG: 8-Hydroxyguanosine
AGE: Advanced glycation end product
IL-1β: Interleukin-1β
CRP: C-reactive protein
HbA1c: Hemoglobin A1c
PD: Probing pocket depth
BOP%: Percentage of bleeding at the probing sites
ELISA: Enzyme-linked immunosorbent assay
