# Supplementary material for: Relationship between oral hypofunction and salivary biomarkers in older adults: a cross-sectional study
Source: BMC Oral Health. 2024 Jul 6;24:766. doi: 10.1186/s12903-024-04556-4 (PMC11227702; doi:10.1186/s12903-024-04556-4)
Supplement: Supplementary file 1 — Additional file 1. Multiple logistic regression analysis with the diagnosis of oral hypofunction as the objective variable. P-value was derived using Multiple Logistic regression analysis. *P < 0.05, OR: odds ratio, CI: confidence interval. Objective variable was the diagnosis of oral hypofunction (Normal oral function = 0, Oral hypofunction = 1). All explanatory other than sex, periodontal stage, diabetes mellitus and hypertension were continuous variables. Model 1 adjusted for age and sex (0 = female, 1 = male). Model 2 adjusted for the variables in model 1 and periodontal stage (0 = stage I+II, 1 = stage III+IV). Model 3 adjusted for the variables in model 1 and diabetes mellitus (1 = positive). Model 4 adjusted for the variables in model 1 and hypertension (1 = positive). [file 12903_2024_4556_MOESM1_ESM.pdf]

Additional file 1 Multiple logistic regression analysis with the diagnosis of oral hypofunction as the objective variable

	Model 1			Model 2			Model 3			Model 4		
Explanatory Variables	OR	95%Cl	P-value	OR	95%Cl	P-value	OR	95%Cl	P-value	OR	95%Cl	P-value
calprotectin	1.004	1.000-1.009	0.048*	1.004	1.000-1.009	0.047*	1.004	1.000-1.009	0.049*	1.00	1.000-1.01	0.040*
8-OHdG	1.294	0.490-3.414	0.603	1.300	0.500-3.382	0.590	1.042	0.372-2.922	0.938	1.10	0.438-2.760	0.840
AGE	1.005	0.994-1.016	0.340	1.005	0.995-1.016	0.331	1.005	0.994-1.016	0.386	1.01	0.995-1.020	0.304

P-value was derived using Multiple Logistic regression analysis. *P < 0.05, OR: odds ratio, CI: confidence interval
Objective variable was the diagnosis of oral hypofunction (Normal oral function = 0, Oral hypofunction = 1).
All explanatory other than sex, periodontal stage, diabetes mellitus and hypertension were continuous variables.
Model 1 adjusted for age and sex (0 = female, 1 = male).
Model 2 adjusted for the variables in model 1 and periodontal stage (0 = stage I+II, 1 = stage III+IV).
Model 3 adjusted for the variables in model 1 and diabetes mellitus (1 = positive).
Model 4 adjusted for the variables in model 1 and hypertension (1 = positive).
