# Supplementary material for: Relationship between oral hypofunction and salivary biomarkers in older adults: a cross-sectional study
Source: BMC Oral Health. 2024 Jul 6;24:766. doi: 10.1186/s12903-024-04556-4 (PMC11227702; doi:10.1186/s12903-024-04556-4)
Supplement: Supplementary file 2 — Additional file 2. Multivariable linear regression analysis with the number of examinations outside the refence range. P-value was derived using Multiple liner regression analysis. CI: confidence interval. Objective variable was the number of examinations outside the reference range. All explanatory variables other than sex, periodontal stage and hypertension were continuous variables. Model 1 adjusted for age and sex (0 = female, 1 = male). Model 2 adjusted for the variables in model 1 and periodontal stage (0 = stage I+II, 1 = stage III+IV). Model 3 adjusted for the variables in model 1 and diabetes mellitus (1 = positive). Model 4 adjusted for the variables in model 1 and hypertension (1 = positive). [file 12903_2024_4556_MOESM2_ESM.pdf]

Additional file 2 Multivariable linear regression analysis with the number of examinations outside the reference range

	Model 1			Model 2			Model 3			Model 4		
Explanatory Variables	Estimate	95%Cl	P-value	Estimate	95%Cl	p-value	Estimate	95%Cl	p-value	Estimate	95%Cl	p-value
calprotectin (n=111)	0.12354	-0.11724, 0.36432	0.31	0.13252	-0.10876, 0.37379	0.29	0.12667	-0.11517, 0.36851	0.30	0.13806	-0.10404, 0.38015	0.26
8-OHdG (n=114)	0,11291	-0.21938, 0.44519	0.50	0.10051	-0.23311, 0.43412	0.55	0.00864	-0.35456, 0.37184	0.96	0.08294	-0.23858, 0.40647	0.61
AGE (n=115)	0.07860	-0.02798, 0.18519	0.15	0.07122	-0.03753, 0.17996	0.20	0.06985	-0.03301, 0.1727	0.18	0.07173	-0.03089, 0.17435	0.17

P-value was derived using Multiple liner regression analysis. CI: confidence interval
Objective variable was the number of examinations outside the reference range.
All explanatory variables other than sex, periodontal stage and hypertension were continuous variables.
Model 1 adjusted for age and sex (0 = female, 1 = male).
Model 2 adjusted for the variables in model 1 and periodontal stage (0 = stage I+II, 1 = stage III+IV).
Model 3 adjusted for the variables in model 1 and diabetes mellitus (1 = positive).
Model 4 adjusted for the variables in model 1 and hypertension (1 = positive).
